# EEG-Neurofeedback as a Potential Therapeutic Approach for Cognitive Deficits in Patients with Dementia, Multiple Sclerosis, Stroke and Traumatic Brain Injury

**DOI:** 10.3390/life13020365

**Published:** 2023-01-29

**Authors:** Irini Vilou, Aikaterini Varka, Dimitrios Parisis, Theodora Afrantou, Panagiotis Ioannidis

**Affiliations:** 2nd Department of Neurology, AHEPA University Hospital, Aristotle University of Thessaloniki, 54621 Thessaloniki, Greece

**Keywords:** EEG neurofeedback, cognitive deficits, memory rehabilitation

## Abstract

Memory deficits are common in patients with dementia, such as Alzheimer’s disease, but also in patients with other neurological and psychiatric disorders, such as brain injury, multiple sclerosis, ischemic stroke and schizophrenia. Memory loss affects patients’ functionality and, by extension, their quality of life. Non-invasive brain training methods, such as EEG neurofeedback, are used to address cognitive deficits and behavioral changes in dementia and other neurological disorders by training patients to alter their brain activity via operant activity. In this review paper, we analyze various protocols of EEG neurofeedback in memory rehabilitation in patients with dementia, multiple sclerosis, strokes and traumatic brain injury. The results from the studies show the effectiveness of the ΕΕG-NFB method in improving at least one cognitive domain, regardless of the number of sessions or the type of protocol applied. In future research, it is important to address methodological weaknesses in the application of the method, its long-term effects as well as ethical issues.

## 1. Introduction

Cognitive deficits, such as memory loss and attention disorders, are very common nowadays because of the aging world population and the numerous neurodegenerative diseases that lead to cognitive impairment. Until recently, the treatment of cognitive deficits was based exclusively on the administration of appropriate medication. However, a deeper understanding of the nature of cognitive deficits combined with advances in technology has led to the development of methods and techniques aimed at improving both cognitive deficits and non-cognitive conditions, in order to improve the quality of life not only for the patients but also for their families.

A popular method is cognitive rehabilitation. Cognitive rehabilitation is a behavioral approach that aims to improve patients’ cognitive deficits, but also to provide assistance to both patients and their families in order to improve their daily lives. It can be carried out with paper and pencil exercises, but also with electronic programs, through which the patient practices various cognitive tasks [1].

Another method is neurofeedback (NFB). NFB is a biofeedback technique for training patients with neurological and psychiatric disorders to change brain activity through operant conditioning [2]. Through this method, the individual learns to enhance and inhibit specific electrophysiological parameters through the process of learning. Modification of the individual’s behavioral response is made possible through feedback and positive reinforcement [3]. EEG-NFB is investigated in patients with depression, post-traumatic stress, schizophrenia, addictions, attention deficit hyperactivity disorder, autism and learning disabilities, and it is correlated with short- or long-term symptom relief.

Neurophysiological factors for NFB training are not clearly defined. It is considered that two types of neuroplasticity are involved: Hebbian plasticity and homeostatic plasticity. Reformation of the neural membrane and synaptic potentiation because of EEG amplitude are characteristics of Hebbian neuroplasticity, while homeostatic plasticity plays the opposite role, in order to stabilize the neuronal activity and limit the expression of the Hebbian type. In any case, the neuronal mechanisms have not been clearly investigated until now [4,5].

There are three types of EEG-NFB: 1. slow cortical potentials; 2. coherence training; and 3. frequency training. The third type of training is the most commonly used and is aimed at modifying the power ratio of frequency bands. The frequency bands are divided into 1. delta, 2. theta, 3. alpha, 4. beta and 5. gamma [6].

These frequencies are used to classify brain oscillations [7]. They are created by the coordinated activity of cells and thus make it possible to communicate between different brain regions in a way that allows the brain to utilize the information it receives and then synthesize it [3]. Additionally, brain oscillations are connected with specific cognitive functions. Theta oscillations are connected with encoding retrieval, while alpha bands are connected with attention. Alpha and gamma oscillations are capable of suppressing factors that can reduce concentration [8].

The most commonly used EEG-NFB protocols for the therapeutic management of cognitive deficits are: 1.EEG-theta/beta ratio; 2. Sensorimotor Rhythm (SMR) (12–15 Hz) [9]. In the first protocol, theta band power (ranging between 4 and 7 Hz) divided by beta band ratio (ranging between 13 and 30 Hz) shows cortical and sub-cortical brain interactions [10]. The second protocol, SMR, includes rhythm with a mean frequency of 10 Hz, and it is recorded over sensorimotor cortices in C3 and C4 [11].

The effectiveness of EEG-NFB in cognitive rehabilitation is still under investigation and is mainly evaluated through comparisons of patients’ cognitive performance before and after their training in various EEG protocols. A review of studies using EEG-NFB as a therapeutic tool for treating cognitive deficits in patients with Alzheimer’s dementia (AD), mild cognitive disorder (MCI), stroke, multiple sclerosis (MS) and traumatic brain injury (TBI) is analyzed below. The novelty of our review lies in gathering findings on the effectiveness of EEG neurofeedback as a therapeutic method in the aforementioned four neurological disorders. Recent studies demonstrating the effectiveness of the method, regardless of whether EEG-NFB is used as a clinical tool or a wearable device, are reviewed.

### Contribution of Our Review

- Review of recent EEG-NFB studies in dementia, multiple sclerosis, strokes and TBI.
- Therapeutic effectiveness of EEG-NFB regardless of how it is applied (clinical use of EEG or wearable device).

## 2. Materials and Methods

To carry out the present study, we searched for EEG neurofeedback studies in patients with dementia, strokes, multiple sclerosis and traumatic brain injury. The literature search was conducted until the end of October 2022. The bibliographic bases used were PubMed, Scopus, ScienceDirect, Cochrane Library and PsycINFO. We used the following key words to search the appropriate studies for our literature review:

1. Neurofeedback;
2. ΕΕG-NFB;
3. Dementia;
4. Alzheimer;
5. MCI;
6. Stroke;
7. Multiple sclerosis;
8. TBI.

We selected the studies based on the following inclusion criteria: 1. surveys conducted during the period 2010–2022; 2. studies that evaluated the effects of EEG-NFB on the cognitive functions of patients with dementia, MS, stroke and TBI; 3. studies in which neuropsychological/cognitive assessments were performed before and after the intervention with EEG-NFB; and 4. scientific papers and case reports.

The exclusion criteria were: 1. scientific conference documents; 2. dissertations; and 3. study protocols.

**Figure 1:**
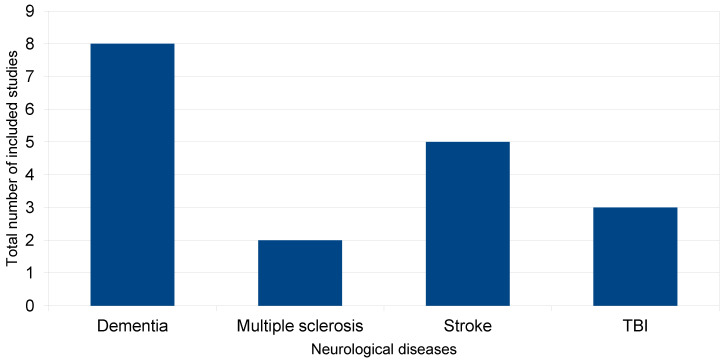
Included studies.

In accordance with inclusion criteria, we selected 8 studies that examined the effects of EEG-NFB in cognitive functions of patients with dementia (2 studies for dementia and 6 studies for MCI, of which 1 study was a case report), 2 studies that also examined the effectiveness of this method in patients with MS, 5 studies for strokes (2 of which were case reports) and 3 studies for TBI (1 of which was a case report). The total number of included studies is presented in the diagram below (Figure 1).

### 2.1. EEG-NFB Protocols Used in Dementia Studies

In 2016, Lujimes et al. (2016) evaluated the efficacy of EEG-NFB in patients with AD. Researchers evaluated patients who followed the NINCDS-ADRDA guidelines. The total sample was N = 10 patients with a mean age of 71.57 ± 6.8 years. Patients underwent neuropsychological assessment with the administration of the Cambridge Cognitive Examination (CAMCOG). All patients were on medication with cholinesterase inhibitors and trained in an EEG-NFB program. They performed 30 sessions, with a frequency of twice a week. More specifically, they were trained to self-regulate different EEG bands on the electrodes of the midline. Each session contained 4 blocks with 5 min breaks. Feedback was given auditorily and visually via movies with a beeping sound and varying contrast. After the completion of the program, a reassessment of cognitive functions was performed, where a 2% improvement in patient performance in CAMCOG was found, while a statistically more significant improvement was in the memory learning subscale [12].

In the same year, Surmeli et al. (2016) conducted EEG-NFB training in 9 patients with AD (Alzheimer Disease) and 11 patients with VD (Vascular Dementia). Patients were on medication with cholinesterase inhibitors and antidepressants. Through special training protocols, they were trained in the self-regulation of different frequency bands on the scalp, performing a different number of sessions based on the individualized program. After 10–96 sessions, there was an improvement in their performance in the Mini-Mental State Examination (MMSE) compared to the initial evaluation, before the beginning of the training program, as well as a decrease in the Clinical Global Impression [13].

Jang et al. (2019) examined the efficacy of EEG-NFB in 5 patients with MCI and a mean age of 66.6 ± 3.5, who were trained in developing strategies to regulate beta frequency bands (12–15 Hz) in F6 (lateral region PFC). Patients participated in 16 sessions that took place over a period of 8 weeks. Before the beginning of the training, a cognitive assessment was performed using the Korean version of Montreal Cognitive Assessment (MoCA) and a computerized neurocognitive battery test (CNSVS). Additionally, hemodynamic changes in the PFC during the working memory test were recorded, and patients completed BDI for depression. Upon completion of the training, patients showed an improvement in MoCA performance and an improvement in complex memory, cognitive flexibility, composite attention, reaction time and executive function [14].

In the same year, Kaufman et al. (2019) investigated the effects of EEG-NFB in an elderly woman with Minor Neurocognitive Disorder. A neuropsychological assessment was performed before and after the training of the patient. The patient participated in a program of 12 sessions with 6 training blocks. She was successively trained in two EEG-NFB protocols (1. SMR rhythm and 2. Alpha Asymmetry). The results showed that the Alpha Asymmetry protocol led to an improvement in memory and sleep, but not in attention and depression [15].

Li et al. (2020) trained 40 patients with an average age of 54.23 ± 5.9 years in self-regulation of alpha and beta power zones. More specifically, this was done over a period of 10 days, divided into two 5-day workouts that included 1 session per day. During one game, there was an alternation between relaxation and concentration, and EEG signals at rest were collected three times: before training, in the second training period and after the second training period. During each session, participants took visual feedback. The result of the EEG-NFB training was increased connectivity between the delta, theta, alpha and beta zones, but no behavioral change was observed [16].

Marlats et al. (2020) included 22 MCI patients in their study. Only 20 patients with a mean age of 76.1 completed the training. They were trained to adjust the SMR upwards, recording it with an electrode from the Cz channel and downwards the theta (4–8 Hz) and beta (12–21 Hz) rhythm. They participated in 30 sessions two or three times a week over a period of 4 months. Patients took visual and auditory feedback via animated graphics. The EEG recording and neuropsychological assessments were performed before the beginning of the training, after its completion and within 30 days after completion, in order to determine whether the result was maintained. These patients showed improved scores on the MoCA test, as well as on the Goldberg Anxiety Scale (GAS) and the Wechsler Adult Intelligence Score IV (WAIS-IV). There was an increase in overall power for theta and alpha frequencies while maintaining changes in the EEG, while scores on the MoCA test returned to baseline [17].

In the study of Lavy et al. (2021), 30 MCI patients with an average age of 71.93 years were divided in a 1:1 ratio into an experimental group, which received neurofeedback training to increase the individual upper alpha zone in the central parietal region, and a control group, which received false neurofeedback with feedback given by random electrodes. The cognitive assessment before and after the completion of the intervention, as well as 30 days after the completion of the intervention, was done with the electronic cognitive assessment battery, NeuroTrax ^TM^. Each session consisted of 10 trials of 3 min, separated by breaks of 10 s, while auditory and visual feedback was given to patients via a beeping sound and balls moving in 3D. The results showed an improvement in performance in memory tasks in the patients of the experimental group. These results were maintained one month after the completion of the training [18].

McLaughlin et al. (2022) applied a BCI (Brain Computer Interface) EEG neurofeedback protocol to 6 patients with mild AD and an average age of 57.66 years. Their goal was to improve patients’ visual attention and language skills through their participation in a 4–7-week program. They were assessed before and after the intervention with the Discourse Comprehension Test and the Digit Span Forward and Backward from WAIS. During the sessions, participants were trained to track a target letter while searching in a 9-letter array for the target letter. The goal was to elicit a P300 signal in response to the target letter, and visual feedback was given to patients. The results of the study showed that all participants with mild AD learned to operate a BCI spelling system with training. Increased frontal power theta was recorded, while beta power was stable [19].

EEG-NFB protocols used in dementia studies are presented in Table 1.

### 2.2. EEG Neurofeedback Protocols Used in Multiple Sclerosis Studies

Keune et al. (2019) investigated the efficacy of EEG-NFB in patients with multiple sclerosis. Their study involved 58 patients with MS (RRMS = 18, SPMS = 35, PPMS = 3) with an average age of 46.70. Prior to the beginning of the training, a neuropsychological assessment was performed on all patients with the short form of the Brief Repeatable Battery, the SDMT test and the Wurzburger Fatigue Inventory. The training had a duration of 2 weeks, and there were 5 sessions. Electroencephalographic activity was recorded at rest with 1 frontal electrode Fz using mastoid reference (M1, M2) during training. Patients were instructed to maintain the theta/beta ratio below the threshold. In each session, resting state EEG was recorded for 2 min, followed by a 4 min block. Visual feedback was given to participants during the sessions. The results showed that patients with slow information processing speed, as derived from SDMT, had an increased theta/beta rate. This rate was reduced during sessions [20].

Kober et al. (2019) conducted a pilot study on 14 patients with RRMS or SPMS with an average age of 38.9 ± 2.2. According to their cognitive performance in the BRB-N battery, patients were divided into responders and non-responders. They were divided equally into groups and participated in 10 NFB sessions within 3–4 weeks at home via a tele-rehabilitation system. The EEG was recorded via semi-dry Ag/AgCI electrodes in the Cz. Electrodes were placed in the right and left mastoid position. Patients were trained to program, and visual feedback was given through sessions. The results showed that the patients who participated in the study showed an improvement in long-term memory and executive functions after completing the training [21].

Table 2 shows the EEG-NFB protocols used in multiple sclerosis studies.

### 2.3. EEG Neurofeedback Protocols Used in Stroke Studies

Cannon et al. (2010) presented the case of a 43-year-old woman after an ischemic stroke in the right hemisphere. The patient received 52 sessions of NFB according to the following pattern: from session 1 to 26, theta (4–7 Hz) and high beta (22–36 Hz) were inhibited and low-beta (13–15 Hz) was rewarded. From session 27 to 32, alpha wave (9–12 Hz) was rewarded. Sessions 33 to 52 included a beta inhibition (16–36 Hz) and slow wave reward (1–15 Hz). Each session lasted for 30 min a day, 2 days a week. The trained regions were P3 and Pz, POZ, T6-T5-Pz, respectively. A symptom checklist (7-point Likert scale) was provided to the patient every 10 sessions. At the end of NFB training, she mentioned improvement of her attention, reaction time and concentration [22].

Mroczkowska et al. (2014) published a case report, in which neurofeedback was used alongside conventional rehabilitation methods for a 53-year-old woman after a hemorrhagic stroke in the left hemisphere. She participated in 10 sessions, 40 min/day, 3 days a week, receiving the C3:C4 (β/τ: SMR/τ) training. The feedback was probably auditory, although it is not clearly described. The results showed an improvement of concentration, visual perception, categorizing and regulation of affect. The Mini-Mental State Examination (MMSE score) was initially 25 points, and at the end of the therapy, it was identified at 28 points. In addition, in the Tools for Optimal Performance States (TOPS assessment) her physiological readiness was calculated at 11 points (3 points pre-assessment), mental readiness at 21 points (6 points pre-assessment) and state of active mental work at 27 points (8 points pre-assessment) [23].

Cho et al. (2015) published a randomized control trial with 27 stroke patients (mean age of 63 years), separated into intervention and control group. In total, 13 stroke patients were in the intervention group and received the same number of conventional rehabilitation sessions as the control group, including NFT training, with a beta-SMR protocol. Each session lasted 30 min, 5 days a week for 6 weeks. Feedback was given through video games. The electrodes were positioned at the C5 or C6 region, and the remaining poles to both ears. NFB training was conducted with the participant’s eyes open, and the rewards were auditory and visual. In addition, four video games were used as stimuli. At the end of the session, statistically significant differences in the motor-free visual perception test (MVPT score) after NFB training, especially in visual discrimination, visual memory and spatial relation, were recorded [24].

Kober et al. (2015) trained 5 groups of participants. In total, 11 stroke patients (mean age of 58.72 years) received SMR-NFB training, 6 stroke patients (mean age of 71.17 years) received upper alpha NFB and 7 stroke patients (mean age of 65 years) received the conventional rehabilitation. In addition, 40 healthy persons also performed NFB training, either SMR protocol (16 persons, mean age of 55.13 years) or upper alpha protocol (24 persons, mean age of 62.63 years). Each group participated in 10 sessions for 4 weeks, receiving audio-visual feedback. After NFB, the SMR patient group and the SMR control group showed significant improvements in verbal short- and long-term memory and in visual-spatial memory (CVLT—California Verbal Learning Test; and VVM2 tests—Visual and Verbal Memory Test). The UA group improved the verbal memory (CVLT test) and the working memory significantly (CBTT backward task, Corsi Block Tapping Test) [25].

Reichert et al. (2016) trained a 74-year-old man with memory deficits, who suffered a stroke due to basilar artery thrombosis. A healthy group of 10 people (mean age = 70.6 years) participated as well. All the participants received 10 sessions of SMR training with visual feedback. In neuropsychological tests, the patient showed significant improvements in memory tasks, such as non-verbal short-term memory and working memory (VVM2 and CBTT backward task). After training, all the participants improved on their non-verbal learning task [26].

For an overview of EEG-NFB protocols in stroke studies, please see Table 3.

### 2.4. EEG Neurofeedback Protocols Used in TBI Studies

Munivenkatappa et al. (2014) trained two patients with a mean age of 15 years and moderate brain injury. Their computer tomography scans showed possible diffuse axonal injury. Both patients participated in 20 sessions of EEG neurofeedback, 40 min/day, 3 days a week for 2 months. The EEG-NFB protocol involved theta (4–7 Hz) and alpha (8–12 Hz) wave frequencies. Pre- and post-neuropsychological assessments (Rivermead concussion symptoms scale and NIMHANS Neuropsychology Battery) showed improvements in mental speed, working memory and retrieval of visual memory [27].

Rostami et al. (2017) published a randomized controlled trial with 13 patients with moderate TBI who were trained in EEG-NFB. Their age was between 15 and 60 years. Eight patients were in the intervention group, participating in 20 sessions of NFB for four weeks. The control group consisted of five patients who participated in the same sessions from the fifth to eighth week of the project. The protocols included beta and alpha coherence methods, the participants had their eyes open and each session lasted 50 min. The electrodes were placed on the FP1-T3 and Cz-Oz regions, respectively. Wechsler Memory Scale (WMS-IV) and Continuous Attention Test (DAUF test) did not show a statistically significant improvement in short-term memory, long-term attention and concentration performance [28].

Arroyo et al. (2021) compared EEG neurofeedback to traditional methods in cognitive rehabilitation. A 20-year-old patient after a brain injury and 3 persons (healthy controls) received NFB training that was based on theta band inhibition. All the participants had 8 sessions of 45 min of NFB training for 2 weeks. The feedback was given visually, and three different visual scenarios were used. The project was continued for 6 weeks in total. The patient had 2 weeks as a rest period, and in the last 2 weeks, he participated in conventional rehabilitation. Attention (BTA task—Brief Test of Attention) was improved not only after NFB, but also after the traditional method. Short-term memory seems to have improved after the conventional rehabilitation (ROCF—Rey Osterrieth Complex Figure; TAVEC—Verbal Learning Test Espana Complutence), while delayed memory shows improvement after NFB training [29].

An overview of EEG-NFB protocols in TBI studies is provided in Table 4.

## 3. Discussion

Our literature review included studies on EEG-NFB interventions in different neurological diseases, such as dementia, multiple sclerosis, strokes and traumatic brain injury. There are studies in which EEG signal is used as a potential predictive tool not only in neurological disorders and rehabilitation but also in sections of daily lives such as sleep stages and driving performance [30,31,32]. In our review, we emphasized the use of EEG signal as a therapeutic tool for cognitive deficits. EEG-NFB is a new and off-label method of cognitive rehabilitation. Thus, the published literature was not very extensive. The majority of studies have shown that EEG-NFB can improve cognitive functions. However, no statistically significant improvement of the same cognitive functions was found in all studies. It was observed that in most of them, there was an improvement in at least one cognitive function. It is worth noting that in the study of Rostami et al. (2017), no statistically significant improvement of cognitive functions was found [28]. Among the studies included in our review, it was found that the number of sessions and frequency of sessions did not appear to affect the effectiveness of EEG-NFB. A different NFB protocol was used in each study, which also did not limit the effectiveness of the method but showed that specific bands are related to different cognitive functions.

In studies by Lujimes et al. (2016) and Surmeli et al. (2016), where training band was determined individually through EEG analysis, an improvement in memory was found through neuropsychological assessment [12,13]. Additionally, in Lavy et al.’s (2021) study, an improvement in memory tasks was found by increasing the individual upper alpha zone in the central parietal region, while in Marlats et al. (2020), an improvement in memory was achieved through individuals training to keep SMR upwards and downwards of the theta (4–8 Hz) and beta (12–21 Hz) rhythm [17,18]. Cannon et al. (2010) presented a complicated pattern of many wave frequencies, while Cho et al. (2015) and Reichert et al. (2016) presented studies with SMR training protocols [22,24,26]. Other studies such as Munivenkatappa et al. (2014) used two different protocols [27]. It is also interesting that the choice of protocol and frequency does not seem to be related to the pathophysiological cause of the cognitive disorder. All of our four sections (dementia, MS, stroke and TBI) used similar protocols, regardless of nature and cause of the disorder. The effectiveness of EEG is emphasized through follow-up studies, which demonstrate the long-term maintenance of EEG-NFB results. Marlats et al. (2020) and Lavy et al. (2020) found that 30 days after the completion of the program, the results of EEG-NFB were maintained, especially in memory (Lavy et al., 2020), while Marlat et al.’s study found that MMSE scores returned to baseline in a follow-up examination [17,18]. In addition, the application of EEG neurofeedback at home creates the conditions for the simpler use of the method, giving patients the opportunity to train their brain in specific frequencies and alter their brain activity, without having to go to the hospital.

In Kober et al.’s (2015) study, it was found that EEG-NFB is effective in different cognitive functions, and it could be used as a tool for cognitive rehabilitation that the patient has the opportunity to use at home receiving guidance from therapist [25]. In addition, side effects of EEG neurofeedback were not reported, and the participants achieved the completion of the study. It is also important and remarkable that healthy participants who took part as control groups (Kober et al., 2015, Reichert et al. 2016) also had improvements of memory and of learning tasks [25,26].

Although these studies were very helpful in drawing conclusions for neurofeedback, it seems that significant information was not reported. Analytical information for the protocols, demographics, the pre- and post-assessment evaluation of cognitive disorders and the long-lasting effects of EEG-NFB were often ignored. In addition, many studies were case reports, and the number of participants was small.

Despite the effectiveness of the method, it is often criticized because there are no specific protocols, no follow-up studies and unclear neurofeedback targets, while there is a lack of testing mobile approaches and a combination of EEG-NFB with other methods [33]. Personalized NFB together with specific protocols would be ideal for the establishment of this method [34].

## 4. Conclusions

EEG-NFB appears to be a potential method that could be used to improve cognitive deficits in patients with neurological disorders. However, there is a necessity to overcome weaknesses in study designs and to investigate further the long-term effects and ethics of this method.

## Figures and Tables

**Table 1:** EEG neurofeedback protocols used in dementia studies.

Dementia	Type of Study	EEG Neurofeedback Protocol	Country
Lujimes et al. (2016) [12]	Pilot study	Determined Individually	The Netherlands
Surmeli et al. (2016) [13]	Experimental study	Determined Individually	Turkey
Jang et al. (2019) [14]	Clinical trial/Experimental study	Beta (12–15 Hz)	Korea
Kaufmann et al. (2019) [15]	Case report	SMR rhythm and Alpha	
Li et al. (2020) [16]	Experimental study	Alpha band (8–13 Hz) and beta (13–30 Hz)/alpha (8–13 Hz) ratio	China
Marlats et al. (2020) [17]	Prospective, randomized controlled trial, single blinded clinical trial	SMR and theta and beta	France
Lavy et al. (2021) [18]	Randomized controlled trial	Alpha (8–10 Hz)	Israel
McLaughlin et al. (2022) [19]	Randomized controlled trial	Frontal power theta and beta	USA

**Table 2:** EEG neurofeedback protocols used in multiple sclerosis studies.

Multiple Sclerosis	Type of Study	EEG Neurofeedback Protocol	Country
Keune et al. (2019) [20]	Randomized controlled trial	Resting state EEG Reduced theta/beta power	Germany
Kober et al. (2019) [21]	Pilot study	SMR (12–15 Hz) theta (4–7 Hz) and beta (21–35 Hz)	Austria

**Table 3:** EEG neurofeedback protocols used in stroke studies.

Stroke	Type of Study	EEG Neurofeedback Protocol	Country
Cannon et al. (2010) [22]	Case study	Combination of frequencies	Great Britain
Mroczkowska et al. (2014) [23]	Case report	β/τ:SMR/τ training	Poland
Cho et al. (2015) [24]	Randomized controlled trial	Beta-SMR protocol	Korea
Kober et al. (2015) [25]		SMR protocol, Upper alpha protocol	Austria
Reichert et al. (2016) [26]		SMR training	Austria

**Table 4:** EEG neurofeedback protocols used in TBI studies.

TBI	Type of Study	EEG Neurofeedback Protocol	Country
Munivenkatappa et al. (2014) [27]		Theta (4–7 Hz) and alpha (8–12 Hz) wave	India
Rostami et al. (2017) [28]	Preliminary randomized controlled trial	Beta and alpha coherence methods	Iran
Arroyo et al. (2021) [29]	Case study	Theta band inhibition	Spain

## Data Availability

Data are available upon reasonable request to the corresponding author.
